# Latina mothers as agents of change in children’s eating habits: findings from the randomized controlled trial *Entre Familia: Reflejos de Salud*

**DOI:** 10.1186/s12966-018-0714-0

**Published:** 2018-10-01

**Authors:** Elva M. Arredondo, Guadalupe X. Ayala, Sandra Soto, Donald J. Slymen, Lucy A. Horton, Humberto Parada, Nadia Campbell, Leticia Ibarra, Moshe Engelberg, John P. Elder

**Affiliations:** 1 Institute for Behavioral and Community Health, School of Public Health, San Diego State University, 9245 Sky Park Ct., Suite 221, San Diego, CA 92123 USA; 2 School of Nursing, University of North Carolina, Carrington Hall, Campus Box #7460, Chapel Hill, NC 97599 USA; 3 Institute for Behavioral and Community Health, 9245 Sky Park Ct, Suite 221, San Diego, CA 92123 USA; 4 School of Public Health, San Diego State University, 5500 Campanile Drive, San Diego, CA 92182 USA; 5 Clínicas de Salud del Pueblo, Inc., 1166 K Street, Brawley, CA 92227 USA; 6 Research Works, 12396 World Trade Dr #313, San Diego, CA 92128 USA

**Keywords:** Latino, Family health, Diet, Health behavior, Nutrition, Family intervention

## Abstract

**Background:**

Few children consume sufficient servings of fruits and vegetables. Interventions aiming to improve children’s dietary intake often target parent level factors, but limited research has examined the mediating role of parental factors on children’s dietary intake. This study examined 10-month follow up data from the *Entre Familia: Reflejos de Salud* (Within the Family: Reflections of Health) trial to investigate (1) intervention effects on children’s dietary intake, both sustained and new changes, and (2) whether changes in mothers’ dietary intake, her parenting strategies, and behavioral strategies to promoting healthy eating in the home mediated changes in children’s dietary intake.

**Methods:**

Participants were 361 Mexican-origin families living in Imperial County, California. Families were randomly assigned to a 4-month dietary intervention or a delayed treatment control group. The intervention was delivered by *promotoras* (community health workers) via home visits and telephone calls. Assessments occurred at baseline, and 4- and 10-months post-baseline.

**Results:**

At 10-months post-baseline, sustained intervention effects were observed on children’s reported intake of varieties of vegetables, with differences getting larger over time. However, differential intervention effects on fast food were not sustained due to significant reductions in the control group compared with smaller changes in the intervention group. New intervention effects were observed on servings of sugar-sweetened beverages. However, the intervention continued to have no effect on children’s reported fruit and vegetable servings, and varieties of fruits consumed. Mother-reported behavioral strategies to increase fiber and lower fat mediated the relationship between the intervention and children’s intake of varieties of vegetables. Mothers’ percent energy from fat and behavioral strategies to lower fat were mediators of children’s daily servings of sugar-sweetened beverages.

**Conclusions:**

This study suggests that a *promotora*-led family based intervention can provide mothers with skills to promote modest changes in children’s diet. Examining the parent related mechanisms of change will inform future interventions on important targets for improving children’s diet.

**Trial registration:**

https://clinicaltrials.gov/. NCT02441049. Retrospectively registered 05.06.2015.

## Background

### Dietary intake among Latino youth

Despite decades of efforts promoting healthy eating patterns, the diets of most US youth do not follow the current USDA (United States Department of Agriculture) dietary guidelines [1]. Research shows that 60% of youth do not eat enough fruit to meet daily recommendations, and 93% of youth do not eat enough vegetables [2]. When considering Mexican American youth, the mean intake of total vegetables in cup-equivalents per 1000 cal among youth aged 2–18 years was 0.56, which is considerably under the target of 1.16 cup equivalent per 1000 cal. Given the health consequences of a poor diet and the opportunity for youth to establish lifelong healthy eating habits, identifying effective parent and family focused interventions in this population should be a public health priority. As Latino youth comprise 22% of all US youth and represent the largest and fastest-growing racial/ethnic minority group in the nation [3], targeted efforts for Latinos are warranted.

### Parent factors associated with healthy eating

Diet-related parenting strategies (e.g., control, reinforcement) play an important role in a children’s diet. A parent’s actions and parenting style can increase or decrease the likelihood that their children will consume healthy foods. For instance, a permissive parenting style, or parenting that is indulgent to children’s food requests with few boundaries, is linked to the consumption of low-nutrient-dense foods [4], reduced intake of fruits and vegetables [5], and increased intake of high calorie beverages [6]. Controlling parenting strategies are associated with a lower intake of and preference for healthy foods in children [7, 8]. Parents pressuring their children to eat healthy foods is linked to a lower intake of fruits [5], while reinforcing in the form of encouragement is linked to increased fruit and vegetable consumption [9].

Parents can also facilitate healthy eating among family members by using behavioral strategies to reduce fat (e.g., use low fat cheese) and increase vegetable intake (e.g., adding vegetables to recipes) in the preparation of foods that are served to family members [10]. Furthermore, studies show that parental dietary intake is highly correlated with children’s intake [6, 9, 11]. Pearson and colleagues conducted a systematic review of family correlates of children and adolescents’ fruit and vegetable intake. The investigators found that parental intake was positively associated with children and adolescents’ fruit and vegetable consumption [9]. However, research is needed to better understand the factors that may explain the concordance of parents’ and children’s dietary intake.

### Parent and family interventions to promote healthy eating among children

Several parent and family-focused interventions have sought to improve children’s dietary intake by changing parenting strategies and behaviors [12–16]. However, few have tested the mechanisms by which interventions impact children’s dietary intake. Spence and colleagues found that higher maternal feeding knowledge and lower use of foods as rewards was the mechanism by which the intervention impacted children’s diet quality defined as intake of fruits and vegetables in children younger than 5 years [16]. Intervention studies targeting the parenting strategies of Latino families have also influenced changes in fruit and vegetable intake among young children [12]. Parents also make decisions about the types of foods served to the family and make choices on how to prepare meals and the types of foods purchased. Examining the mediating role of parenting strategies and other parental factors (e.g., dietary behavioral strategies) in changing children’s dietary intake is important because they become the targets for health behavior change in health promotion interventions [17]. Given findings from a recent systematic review that most (about 70%) published research has focused on younger children (4–8 year olds), studies are needed to better understand parental factors that may be effective in improving dietary intake among older children [18].

### Description of current study

This study investigated the effects of *Entre Familia: Reflejos de Salud (*Within the Familiy: Reflections of Health), a family-based intervention, on children’s reported intake of fruits (daily servings and monthly varieties), vegetables (daily servings and monthly varieties), sugar-sweetened beverages (daily servings), and fast food (weekly frequency). We examined baseline to 10-month dietary changes and investigated the mediating role of parents’ behavior (parenting and dietary behavioral strategies), as well as parents’ dietary intake on changes in children’s dietary intake. This study builds on a previous analysis demonstrating that the 4-month intervention significantly decreased fast food intake, and trends were observed in the varieties of vegetables consumed by children [19]. In addition, we found 4-month intervention effects on mothers’ reported consumption of vegetables, as well as behavioral strategies to increase fiber and decrease fat intake [20]. In this study, we examined whether the intervention helped to sustain the changes observed at 4-months on varieties of vegetables consumed and weekly consumption of fast food, as well as whether it resulted in new improvements in children’s servings of fruits, vegetables and sugar-sweetened beverages, and varieties of fruits consumed at 10 months. Because the intervention targeted dietary intake, parenting strategies and food related behaviors in the home, we examined whether mothers’ dietary intake (i.e., daily servings of fruits and vegetables, percent calories from fat), her parenting strategies (i.e., control, reinforcement, etc.), and dietary behavioral strategies to increase fiber and reduce fat, mediated changes in children’s diet.

## Methods

### Aim, study design and setting

*Entre Famili*a was a two-group randomized controlled trial. We randomly assigned 50% of the families to a 4-month intervention and 50% to a delayed treatment control group. We collected child-reported dietary intake and mother-reported parenting strategies, dietary intake, dietary behavioral strategies, and household/family characteristics (e.g., family cohesion) at baseline, immediately post-intervention (4-months) and 6 months later (10-months post-baseline).

This study was conducted in Imperial County, California, the southern-most county in California with a population of approximately 174,000 residents; 82.7% of Imperial County residents are Latinos and mostly of Mexican origin [21]. In 2011–2012, 78% of the adult population in Imperial County was overweight or obese compared with 60% for the state of California [22]. Approximately 24.3% of families were living under the federal poverty level, compared to 13.5% in the US. The community is considered a rural community.

### Participants

Between May 2009 and February 2011, we recruited families through a number of mechanisms including tables at community health fairs, advertisements in newspapers and other weekly circulars, and letters mailed to potentially eligible families from our partner agency, *Clínicas de Salud del Pueblo, Inc.*, a federally qualified health center with 12 clinics. At events and other face-to-face contact opportunities, families were encouraged to sign up for possible participation. Where possible, mothers and children were screened on the spot by trained, bilingual recruitment staff. If not, interested families, specifically the mothers, were called to learn more about the study, and if interested, screened for possible inclusion. Flyers and other print materials provided information on a number to call if interested. The same process was used for these families. Families were eligible to participate based on the mother’s eligibility criteria: at least 18 years of age, resident of Imperial County, has at least one child between the ages of 7 and 13 years, self-identifies as Latina, reads and speaks Spanish, and lives in the same household as her child and husband/partner for at least 4 days of the week. Families with a member on a medically-prescribed diet and/or planning to move outside of Imperial County during the study period were excluded. If interested and eligible, written consent from the mother and written assent from the child were obtained in order to participate; in a subsample of 25% of the families, consent was also obtained from the husband/father to participate in the evaluation cohort assessing his diet and parenting strategies (data not reported here) [23].

The study Evaluation Coordinator used simple randomization procedures (e.g., computer generated random numbers) to randomly assign eligible families to the intervention or delayed treatment control group as soon as the mother, the selected child, and the husband/father (among 25% of families) completed their baseline assessments. Only mothers and their children are included in the present analyses. Importantly, all family members assigned to the intervention group were invited to participate in the intervention.

### Intervention

*Entre Familia* was a family-based intervention that targeted promotion of fruit and vegetable intake through the modification of parent health behaviors, parenting strategies and other family and household influences on diet. Intervention development [24] was based on the integration of two theoretical frameworks; one focused on behavior change (Social Cognitive Theory [25]) and the second focused on family processes (Family Systems Theory [26]). Details of the intervention are available elsewhere [20]. Table 1 illustrates how these two theories were integrated to design an intervention that focused on the use of positive reinforcement and effective family communication.

**Table 1:** Example of behavior change and family system theories integrated into a home visit

Theoretical construct/Behavior change taxonomy	Goal(s)	Watch and discuss DVD	Targeted Behavior	Fun activity
Goal setting (behavior)	Health behavior change through family change	Family meals	Add more vegetables to dinner	Broken hearts: How positive and negative communication affects us
Social incentive/reward	Effective family communication and use of positive reinforcement	Using food as a reward	Identify reinforcing family activities	

#### Promotora training

Consistent with other family-based interventions the authors have developed [27–29], *Entre Familia* was delivered by *promotoras*, also known as community health workers. *Promotoras* are trusted members of a community [30], and are trained to provide the type of support families need, including appraisal support that meaningfully ties in contextual challenges of behavior change (e.g., challenges with controlling portions when eating out). Six *promotoras* were hired by *Clínicas de Salud del Pueblo, Inc,* a federally qualified health center in Imperial County, California, and trained to deliver the intervention. Most had served previously as *promotoras* and had experience working with families on topics unrelated to healthy eating (e.g., teen pregnancy). The *promotoras* received 80 h of training. The training involved both didactic and skill-building opportunities, including modeling of home visit intervention delivery and opportunities to practice and receive feedback on their delivery. Among the topics that received the most attention during the training was how to provide social support to families and how to engage families in effective family communication to establish weekly behavior change goals (additional information on the training is available elsewhere [24].

#### Intervention delivery

Families randomly assigned to the intervention group were contacted by a *promotora* within a week of their baseline assessment and received a welcome letter. On the first phone call, the *promotoras* introduced themselves to the family and scheduled the first home visit. Given our primary focus on mothers, the *promotora* asked to speak with the mother; however, if she was not available and if her husband/father lived in the home, the *promotora* would instead ask to speak with the husband/father.

The intervention was delivered over a 4-month period, with contact occurring more frequently initially and tapering over time. This design element helped promote autonomy and support seeking from existing social network members, and created a socially supportive environment for sustained behavior change. Thus, the first home visit occurred 1–2 weeks after the baseline assessment, with subsequent home visits occurring weekly for the first 2 months (eight visits), biweekly for the third month (2 home visits, plus two biweekly telephone calls), and once during the fourth month (1 home visit plus one telephone call). The total planned dose was 11 home visits at 90–120-min/each and four telephone calls at 15–30-min each (including introductory telephone call). We scheduled home visits to maximize the involvement of all family members, especially the mother, selected child, and husband/father (if present). The home visits were facilitated by the *promotora* who used, among other materials, a 9-part DVD series, based on the concept of edutainment that depicted a typical Mexican-origin family struggling to engage in healthy eating and the sources of influence on this behavior. In process evaluation findings examining predictors of changes at 4-months, satisfaction with the DVD series was associated with changes in behavioral strategies to increase fiber as reported by the mothers [31]. *Promotora* materials were supplemented with a workbook containing the objectives of each home visit, key points from the DVD episode, and behavior change tools consistent with Michie et al., and Bandura including goal setting and self-monitoring forms [25, 32]). The *promotora* home visits followed a standard protocol. The *promotora* reviewed the previous week’s homework, watched one DVD episode, delivered a mini-presentation on the topic of the day, and facilitated family activities including helping the family set a weekly goal and reviewing the next homework assignment. During brief telephone calls on intervening weeks, *promotoras* problem-solved barriers to meeting the goals of the week.

#### Control group

Once randomized to this group, families in the delayed treatment control group received a letter in the mail indicating their group assignment and a reminder about the upcoming 4- and 10-month post-baseline assessments. Families in the delayed treatment control group received the intervention materials only (DVD and workbook) after completing the 10-month assessment protocol, with an explanation of how to use the materials. They received no contact with a *promotora*.

### Evaluation

Bilingual and bicultural research assistants, blinded to study group, collected data at all three time points from mothers and children at their homes or other private place. All scales were available in Spanish and have adequate psychometric properties in Latino/Hispanic communities. The mother and child participated in separate interviews, followed by measurement of their height and weight to calculate body mass index.

#### Child intake (child report)

##### Daily fruit and vegetable intake

Daily cups of fruits and vegetables was measured with the National Cancer Institute’s Food Attitudes and Behavior Survey 2-item assessment [33] that asks the following questions: “About how many cups of fruit, including 100% fruit juice, do you eat or drink each day?” and “About how many cups of vegetables, including 100% vegetable juice, do you eat or drink each day?” Children were given food models to estimate their intake as previously done with children. The dependent variables used in the models were daily cups of fruits and daily cups of vegetables.

##### Monthly varieties of fruits and vegetables

The varieties of fruits and vegetables consumed was assessed by asking children to identify any of the 30 fruits or 44 vegetables consumed in the past month using the name and a picture of each fruit and vegetable [34]. The dependent variables used in the models were monthly varieties of fruits and monthly varieties of vegetables.

##### Daily servings of sugar-sweetened beverages (SSB)

Daily servings of SSBs was obtained using a modified item from the 5-item subscale from the Youth/Adolescent Questionnaire: “On a typical day, how many cans or glasses of regular soda, Kool-aid, Tampico or punch do you drink?” Responses range from none to seven [35].

##### Weekly fast food consumption

Weekly fast food consumption was assessed by one item on the number of days the children ate fast food in a typical week [36].

#### Parent intake (parent report)

##### Daily servings of fruits and vegetables

Mothers’ daily intake of fruits and vegetables in the past month was assessed with the 19-item National Cancer Institute Fruit and Vegetable All-Day Screener [37]. Mothers reported their frequency of consuming various types of fruits and vegetables with response options ranging from never to 5 or more times per day. Each frequency question was followed by a question on the typical amount consumed. Food models were provided to assist with estimating portion sizes. Continuous scores representing total daily servings of fruits and total daily servings of vegetables consumed in the past month (excluding French fries, potatoes and beans/legumes) were calculated from the screener results.

##### Percent energy from fat

The percent of energy consumed from fat was measured with the National Cancer Institute’s Multifactor Fat Screener [38]. Mothers reported how often they consumed 15 high- and low-fat foods in the past 12 months. Responses were converted to the number of times per day by standardizing frequency responses to their midpoint and then multiplying them by a weighted score that was based on mother’s age for the portion sizes of each food.

##### Monthly varieties of fruits and vegetables

The varieties of fruits and vegetables were assessed by asking mothers to identify any of the 30 fruits or 44 vegetables consumed in the past month using the name and a picture of each fruit and vegetable. Summary scores represented the total varieties of fruits and total varieties of vegetables consumed in the past month.

#### Parenting behaviors (parent report)

##### Parenting strategies

Diet-related parenting strategies were measured with the Parenting Strategies for Eating and Activity Scale (PEAS; [39]). The PEAS was modified for the *Entre Familia* study by removing the 10 physical activity items, adding a new subscale assessing permissiveness parenting strategies, and adding items to the reinforcement and discipline subscales for diet [24]. The 23-item, modified PEAS assessed diet-related parenting strategies across 6 subscales (baseline internal consistency score): monitoring (5 items, α = .86), reinforcement (4 items, α = .65), permissiveness (4 items, α = .78), limit setting (2 items, α = .62), control (5 items, α = .74), and discipline (3 items, α = .74). A sample question of monitoring was: How much do you keep track of the high fat foods your child eats? Response options were on a 5-point Likert scale ranging from never to very often. The mean score for each subscale represented the frequency of using each parenting strategy.

#### Parent report dietary behavioral strategies

##### Behavioral strategies to increase fiber intake

Dietary behavioral strategies to increase fiber intake were assessed using an 11-item scale pertaining to the frequency of engaging in behaviors such as purchasing fruits for snacks [40]. A sample item included: How often did you buy fruits for your meals or snacks in the past month? Response options were on a 4-point Likert scale ranging from never to always with higher scores indicating more frequent use of the behavioral strategy (α = .71).

##### Behavioral strategies to decrease dietary fat intake

Dietary behavioral strategies to decrease fat intake were measured with 19 items pertaining to the frequency of engaging in fat avoidance behaviors such as sharing a high-fat food during a meal [40]. A sample item included: How often did you “Use skim or 1% milk instead of regular or 2% milk in cereal” in the past month? Response options were on a 4-point Likert scale ranging from never to always with higher scores indicating more frequent use of the behavioral strategy (α = .76).

#### Demographic information and covariates

Demographic information collected from mothers included race, education (completed high school or more versus less than high school), marital status (married/living as married versus single, divorced, or widowed), number of children living in the household, employment status (coded as employed/self-employed vs. unemployed/homemaker/retired/unable to work), food assistance including the Special Supplemental Nutrition Program for Women, Infants and Children (WIC) or the Supplemental Nutrition Assistance Program (SNAP) (yes vs. no), home ownership (yes/no), and country of birth (foreign-born vs. US-born). Poverty was calculated by taking into account the family’s income and dividing it by the number of family members dependent on the income; this variable was then categorized as above or below poverty status based on US Census Bureau 2010 thresholds. Parents reported on the children’s age, gender, and country of birth.

### Data analyses

#### Outcome analyses

All analyses were based on the intention-to-treat approach. Each outcome was examined using mixed effects models for normal outcomes (SAS Proc Mixed) or generalized linear mixed effects models for non-normal outcomes (SAS Proc Glimmix). For non-normal outcomes, appropriate error distribution and link functions were chosen according to the type of outcome. Models accounted for repeated measures over 4 and 10 months and adjusted for baseline. Thus, although a participant may have data missing at 4 or 10 months, data available at nonmissing time points were included in the analyses. Terms in the model included a group indicator (intervention vs control), time (4 vs 10 months) and the group by time interaction. In addition, all models adjusted for mother’s race (all identified as Latina but could be of different races), education and marital status. Based on the interaction term, an F test evaluated the group effect at 10 months. All analyses were carried out at the .05 level of significance.

#### Mediation analyses

Our mediation analyses followed procedures outlined by MacKinnon, Fairchild and Fritz [41] and MacKinnon, Fritz, Williams and Lockwood [42]. Mediators are intervening factors that are amenable to change that explain the relationship between the intervention and the outcomes of interest. As such, the intervention is hypothesized to change the mediator, which in turn changes the outcome. Three regression models were fitted yielding the necessary parameter estimates and standard errors. First, the intervention effect was examined on each dependent variable as reported in this paper. Second, the intervention effect was examined on selected sets of variables consisting of changes in mother’s dietary intake (e.g., daily servings of fruit; percent energy from fat) and parent health behaviors (behavioral strategies to increase fiber; behavioral strategies to lower fat); and parenting strategies as reported by the mother. Based on the results of these analyses, outcomes and potential mediators were selected for further evaluation. Finally, for each outcome, the intervention effect and each potential mediator were included in the same model. All models accounted for repeated measures and adjusted for the same set of covariates. As described by MacKinnon et al. [41], the mediated effect is the result of the product of the unstandardized regression coefficient of the intervention effect in model 2 (coefficient a) and the unstandardized coefficient of the potential mediator in model 3 adjusted for the intervention effect (coefficient b). This product, ab, is usually assumed to be normally distributed and its significance is often evaluated using Sobel’s test. However, ab is usually highly skewed and does not follow a normal distribution. MacKinnon, Fritz, Williams and Lockwood [42] developed software that provides more accurate asymmetric confidence limits for the product than that provided by Sobel’s test. A significant mediated effect at a level of significance of .05 is determined if the confidence interval does not include 0. The software, PRODCLIN, is available as a SAS macro.

## Results

### Participants and retention of dyads

**Fig. 1:**
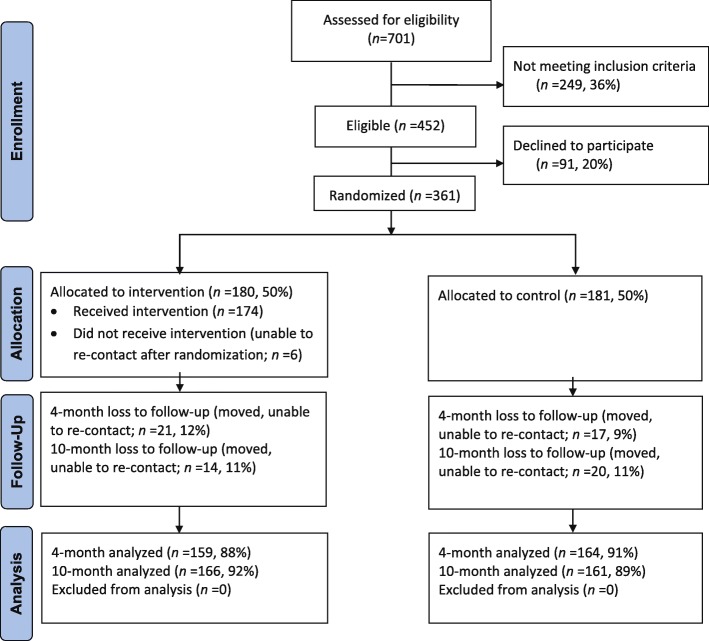
Entre Familia Consolidated Standards of Reporting Trials (CONSORT) flow diagram. Based on results from the Entre Familia: Reflejos de Salud study, carried out in Imperial County, California, USA between 2007 and 2012

Figure 1 shows the CONSORT flow diagram for *Entre Familia* study (full study CONSORT checklist is available as a supplementary document). Enrollment of 361 mothers was based on initial power calculations aimed at mean changes of 0.5/day servings of fruits and vegetables; calculations indicated at least 150 mothers needed per group for 90% power.

Of the 361 families enrolled, 34 families (11%) were lost to follow up at 10 months in both groups. Retention rates at 10 months were 92% and 89% for intervention and control groups, respectively. All baseline evaluation measures were completed for mothers and children as a dyad pair. Follow-up measures at 4-months and 10-months were mostly completed as a dyad pair (4-months: 3 children did not complete measures where the mother did, 1 child did complete measures where the mother did not; 10-months: 2 children did not complete measures where the mother did).

Table 2 shows the demographic characteristics of Latino mothers and their children. With the exception of marital status (control group had significantly more single mothers, *p* < .05), there were no demographic differences between mothers in the intervention and control groups. The majority of Latina mothers were married (94%) and foreign-born (82%). About half completed high school (49%) and were on food assistance (52%). Less than half of the mothers were employed (35%) and owned a home (43%). The children were, on average 10 years old, approximately half were female, and approximately a fifth were foreign born.

**Table 2:** Demographic characteristics of participating Latina mothers and their children (*N* = 361)

	Total sample Mean (SD) or % (*n*)	Intervention (*n* = 180) Mean (SD) / % (*n*)	Control (*n* = 181) Mean (SD) / % (*n*)	Sig.
Parent/home characteristics				
Mean age	38.5 (7.9)	38.4 (8.1)	38.6 (7.8)	n.s.
% married/living as married	94% (338)	96% (173)	91% (165)	*p* ≤ .05
Mean # of children in home	2.00	2.0	2.0	n.s.
% completed high school	49% (175)	49% (89)	48% (86)	n.s.
% employed	35% (125)	38% (69)	31% (56)	n.s.
% on food assistance	52% (186)	54% (96)	50% (90)	n.s.
% own a home	43% (154)	45% (80)	41% (74)	n.s.
% foreign born	82% (295)	78% (141)	85% (154)	n.s.
Child characteristics				
Mean age	10.0 (1.9)	10.0 (1.8)	9.9 (2.0)	n.s.
% female	50% (181)	48% (86)	53% (95)	n.s.
% foreign born	19% (68)	21% (37)	17% (31)	n.s.

### Primary analyses

Table 3 compares the intervention and control groups on children’s dietary outcomes at 10 months. Significant differences were found on monthly varieties of vegetables (*p* = 0.03) where the intervention group mean was 12.6 compared to 11.3 in the control group. Daily servings of sugar-sweetened beverages was also significant (*p* = .02) where the intervention group had a lower average of drinks (M = 1.02) compared to the control group (M = 1.38). Table 3 also compares the parenting strategies between intervention and control groups at 10 months. There were no group-by-time interactions on parenting strategies.

**Table 3:** Group comparisons on children’s outcomes and mother-reported parenting strategies at 10-month post-baseline

	Intervention		Control		*P*
	M	SE	M	SE	
Children’s reported intake (outcomes)					
Daily cups of fruits	1.78	.10	1.86	.10	.58
Daily cups of vegetables	1.17	.07	1.12	.07	.60
Monthly varieties of fruits	10.4	.35	9.9	.35	.29
Monthly varieties of vegetables	12.6	.42	11.3	.42	.03
Daily servings of sugar-sweetened beverages	1.02	.10	1.38	.10	.02
Days per week consuming fast food^a^	1.02	.07	1.10	.07	.44
Mother-reported parenting strategies (mediators)					
Control^a^	2.76	.06	2.89	.06	.11
Reinforcement^a^	3.71	.06	3.64	.06	.35
Monitor	3.21	.08	3.11	.08	.31
Permissiveness	2.74	.08	2.66	.08	.48
Discipline	2.24	.08	2.07	.08	.11
Limit Setting^a^	3.71	.08	3.75	.08	.72

Means were adjusted for baseline. Analyses controlled for mother’s race, WIC status, and marital status

^a^Significant group differences observed immediately after intervention (4 months)

Note: All other potential mediators (changes in mother’s intake and mother-reported family home environment variables) were analyzed as part of Horton et al., paper [43]

Possible mediators and child outcomes were selected for mediation analyses (Table 4). based on the results shown in Table 3 and the mother’s 10-month outcome paper [43], indicating that the intervention improved mothers’ daily servings of fruits (*p* < .05), mothers’ percent energy from fat (*p* < .01), behavioral strategies to increase fiber (*p* < .001) and behavioral strategies to lower fat (p < .001). Behavioral strategies to increase fiber and to lower fat both mediated the effects of the intervention and children’s monthly varieties of vegetables. Furthermore, mothers’ percent energy from fat and behavioral strategies to lower fat mediated the relationship between intervention effects and children’s daily servings of sugar-sweetened beverages.

**Table 4:** Group intervention mediation analyses for selected factors on selected children’s outcomes

Outcome	Mediator	a	b	ab (95% CI)	% mediated
Child monthly varieties of vegetables	Mother daily serving of fruits	0.19	0.10	0.02 (−0.03, 0.09)	1.50
	Mother % energy from fat	−0.94	− 0.07	0.07 (− 0.02, 0.19)	5.50
	Behavioral strategies to increase fiber	0.17	1.43	0.24* (0.09, 0.44)	19.50
	Behavioral strategies to lower fat	0.23	1.03	0.23* (0.04, 0.46)	18.90
Child daily servings of sugar-sweetened beverages	Mother daily serving of fruits	0.19	−0.10	−0.02 (− 0.06, 0.01)	9.60
	Mother % energy from fat	−0.94	0.03	−0.03* (− 0.07, − 0.01)	15.30
	Behavioral strategies to increase fiber	0.17	0.02	0.00 (−0.03, 0.04)	1.80
	Behavioral strategies to lower fat	0.23	−0.24	−0.05* (− 0.11, − 0.01)	26.50

*p < .05

*CI* Confidence Interval

a: Intervention effect on mediator

b: Mediator effect on outcome adjusting for intervention

ab: mediated effect

% mediated: proportion of the absolute total effect that is mediated

## Discussion

Several family-based interventions have targeted children’s dietary intake with mixed results [44]. This study examined the longer-term effects of a family-based intervention on children’s dietary intake, and the extent to which mothers’ health behaviors and parenting strategies mediated changes in children’s diet. We found that a family-based intervention delivered by *promotoras* sustained increases in the monthly varieties of vegetable consumed by children and new reductions in their intake of sugar-sweetened beverages. These findings are consistent with other research that showed changes in fruit/vegetable and high fat/high-sugar intake following participation in a parent and child intervention [45]. Improving the varieties of vegetables consumed by children has the potential to change food preferences with life-long implications on dietary behaviors [46].

This study showed that targeting specific parenting behaviors related to dietary intake helped children increase the varieties of vegetables consumed and decrease the intake of sugar-sweetened beverages. More specifically, mothers’ behavioral strategies to increase fiber and lower fat mediated the association between the intervention and children’s varieties of vegetables consumed. Similarly, mothers’ behavioral strategies to lower fat mediated the association between the intervention and children’s decreased consumption of sugar-sweetened beverages. It may be that the steps that the mothers took to provide healthier beverage options in the home (e.g., less fat in the milk provided to the children) which was addressed in the intervention, included removing sugar-sweetened beverages, These findings suggest that an important target for family-based interventions is providing parents with the skills to prepare foods low in fat and high in fiber such as modifying the preparation of commonly used foods and recipes, and/or replacing unhealthy foods with alternatives.

Contrary to expectations, the intervention did *not* impact parenting strategies related to children’s diet. As such, we were unable to examine these factors as mediators. These findings are in contrast to previous research involving *promotoras* as agents of change. In a randomized controlled trial that involved Latino parents in the promotion of healthy eating and physical activity among young children (K-2nd grade), Crespo et al. found that increased parental monitoring, reinforcement, and control strategies of their child’s diet and physical activity were related to subsequent changes in young children’s consumption of fruits and vegetables [12]. It may be that interventions that target parenting strategies associated with children’s dietary intake are more effective among families that include young compared to older children given that parents are likely to have a stronger influence on younger versus older children’s health behaviors. It also may be that the *promotoras* did not effectively target parenting strategies associated with children’s dietary intake.

Another reason for the lack of concordance between ours and previous findings may be that our measure of diet-related parenting practices (PEAS) is more sensitive with parents who have younger children. PEAS was developed with children who were recruited from kindergarten through 2nd grade, an age where parents may be more able to monitor and reinforce their children’s health behaviors than those of older children. Consistent with this interpretation, a recent study tested the factor loadings of the PEAS scale involving Hispanic mothers of daughters ages 9–14 and found that the originally PEAS scale developed with young children needed to be modified when used with children of this age group [47]. Findings showed that the items comprising these three factors (limit setting, monitoring, and discipline) did not have the same factor loadings identified in the original scale.

The current study suggests that the involvement of *promotoras* in a family intervention is a culturally sensitive and effective approach to increasing vegetable varieties and decreased daily servings of sugar-sweetened beverages consumed. There are a few reasons why this may be the case. Latino families may be more open to the suggestions of *promotoras* given that they are members of the same community. Also, Latino families may find that the information shared by the *promotoras* was pertinent to them given the likelihood that the *promotoras* may have highlighted health information and behavioral strategies most relevant to the participating families. The current study is innovative in that the *promotoras* used culturally tailored videos that showed a common challenge that parents encounter in promoting healthy eating among family members. These situations were designed to be “points of discussion” to stimulate a conversation between family members and the *promotora* which facilitated behavioral skill development [40].

### Limitations and strengths

There are a few limitations worth mentioning. Like other self-report measures, our measures of dietary intake and behavioral change were subject to social desirability. The use of dietary assessments like the inclusion of biomarkers to verify self-reported dietary intake would be preferred [48]. Similarly, as we have noted previously, possible demand characteristics, such as wanting to show the positive effects of a *promotora*-delivered intervention, may explain the positive changes observed among those in the intervention group compared with the control group. We minimized bias by involving trained and blinded evaluation staff, who were separate physically and administratively from the *promotoras*, to collect the data [20]. Another limitation is the involvement of a convenience and homogenous sample limiting the generalizability of the results. Furthermore, there were a number of children’s dietary outcomes that were not influenced by the intervention which may be due to the fact that the primary target was mother’s dietary intake or the dietary changes observed in the children were spurious. In addition, children in this sample reported consuming almost two cups of fruits per day which is consistent with dietary guidelines, introducing the possibility of a ceiling effect. Despite these limitations, the study has several strengths. The children who participated in the study reported their dietary intake, thereby limiting social desirability by the parent. Another strength is that the intervention influenced dietary intake 6 months following the completion of intervention activities, adding to the limited number of studies that examine the longer-term effects of dietary interventions. Also, the study included a rural community that is at high risk for obesity [49]. Furthermore, the current study adds to the limited research in understanding the mechanisms of change that may occur in family based interventions targeting children’s eating practices.

## Conclusions

In conclusion, the current study used an innovative approach to target parent level factors associated with children’s dietary intake. The intervention did impact one of the biggest contributors of obesity among youth, the intake of SSB [50]. Engaging children and their parents in interventions has the potential to change long-term habits associated with the risk for obesity, an important consideration given the high rates of obesity among Latino youth [51]. The current study adds to the limited number of family-based interventions targeting youth.
